# Giant Third-Order Nonlinear Response of Mixed Perovskite Nanocrystals

**DOI:** 10.3390/ma15010389

**Published:** 2022-01-05

**Authors:** Aya M. Abu Baker, Ganjaboy S. Boltaev, Mazhar Iqbal, Mikhail Pylnev, Nasser M. Hamdan, Ali S. Alnaser

**Affiliations:** 1 Department of Physics, American University of Sharjah, Sharjah 26666, United Arab Emirates; aabubaker@aus.edu (A.M.A.B.); miqbal@aus.edu (M.I.); nhamdan@aus.edu (N.M.H.); 2 Institute of Ion Plasma and Laser Technologies, Uzbek Academy of Sciences, Tashkent 100125, Uzbekistan; 3 Centre for Advanced Materials Research, University of Sharjah, Sharjah 27272, United Arab Emirates; mpylnev@sharjah.ac.ae

**Keywords:** ultrashort pulses, mixed perovskite, third harmonic generation, two-photon absorption, laser–matter interactions

## Abstract

Mixed (FAPbI3)_0_._92_(MAPbBr3)_0_._08_ perovskite thin films exhibit strong nonlinear optical responses, rendering them promising candidates for applications in photonics and optical communications. In this work, we present a systematic study on the ultrafast third-order nonlinear optical processes in mixed perovskite nanocrystals (NCs) by exploring the generation of third harmonic radiation and giant two-photon absorption-based photoluminescence (PL) when excited by femtosecond laser pulses of a 1030 nm central wavelength. A comparative analysis of the coherent third harmonic generation in the thin-film-containing perovskite nanocrystals has shown a 40× enhancement of the third harmonic signal compared to the signal generated in the pure quartz substrate. The cubic dependence of the third-nonlinear optical response of the (FAPbI_3_)_0_._92_(MAPbBr_3_)_0_._08_ perovskites on the intensity of the driving radiation was identified using broadband 38 femtosecond driving pulses. The positive nonlinear refractive index (*γ* = +1.4 × 10^−12^ cm^2^·W^−1^) is found to play an important role in improving the phase-matching conditions of the interacting pulses by generating a strong third order harmonic. The giant two-photon absorption (TPA)-assisted PL peak was monitored and a blue shift of the PL was obtained in the higher intensity range of the laser pulses, with the absorption coefficient β estimated to be~+7.0 cm·MW^−1^ at a 1030 nm laser wavelength.

## 1. Introduction

Hybrid organic–inorganic lead halide perovskites of the form APbX_3_, where the A-element generally stands for organic cations such as CH_3_NH_3_^+^ or HC(NH_2_)^+2^, whereas the X-component stands for the anions of halogen (I^−^, Br^−^ or Cl^−^), are attracting great interest due to their high conversion efficiency, which exceeds 25%. The conversion efficiency of perovskite solar cell is defined as the ratio of the electricity generated from a cell and the solar power [1]. Perovskite materials have also proven to be excellent nonlinear optical (NLO) materials due to their optical nonlinear response and broad spectral absorption, which makes them promising candidates for photonics and optoelectronics applications, such as optical data storage and as sources for ultrafast optical signals, and higher-order harmonic generation [2,3,4]. The main component of the mixed [(FAPbI_3_)_1−x_(MAPbBr_3_)_x_] perovskite-like formamidinium (FA)-based perovskites (FAPbI_3_) has a bandgap energy (E_g_) of 1.47 eV with a conversion efficiency exceeding 20% [5]. Meanwhile, the methylammonium lead bromide (MAPbBr_3_) has a wider bandgap energy of around 2.00 eV [6]. The mixture of MAPbBr_3_ with FAPbI_3_ as an active layer for light absorption had shown special characteristics that are vital not only for the improvement of the conversion efficiency but also for tuning the optoelectronic properties of light-harvesting materials included in the perovskite’s structure [7]. Mixed-cation mixed-halide perovskites have special compositional engineering that enhances both the stability and conversion efficiency of the device [8]. Moreover, due to its high stability in optoelectronics devices, mixing FAPbI_3_ and MAPbBr_3_ has been the focus of a large number of studies that aim towards extracting the light absorption coefficient of such a mixture. Previous studies have shown the tolerance of single-crystal perovskite (APbX_3_) to the mixing of the organic components: methylammonium (MA) and formamidinium (FA) cations in the A-site of the (APbI_3_) perovskite structure [9]. It was shown that the linear optical properties of perovskites can be tuned by changing the halide anion X-site of the lead halide perovskite [7,10], which would result in changing the bandgap and in obtaining a tunable photoluminescence (PL) emission as a function of the driving radiation wavelength.

Thin film lead-based halide perovskites are of particular interest due to their strong nonlinear responses (third-order nonlinearities) and potential applications in photovoltaic devices [11], light-emitting diodes [12], and nonlinear optics (NLO) for the generation of coherent radiation through converting the frequency of mid-infrared laser radiation [13]. The third-order nonlinear response has an influence on the generation of the third harmonic and optical Kerr effect [14]. Investigating the third-order nonlinear response of perovskite targets has been performed through the generation of third-order harmonic emission [15] and sensitive Z-scan measurements [16]. Recently, enhanced third harmonic generation of tunable femtosecond laser pulses in CsPbBr_3_ perovskite nanocrystals containing thin films was reported by Bhattacharya et al. [17], where such enhancement was attributed to the crystalline structures of the perovskite. Enhancement of the third-order nonlinear response can play role under resonantly-excited conditions, where the wavelength of the laser pulses corresponds to the absorption band of the medium [18]. It was shown that, in the case of resonant excitation of perovskite, one-photon-excited inter-band free carrier absorption can be dominant over the mechanism responsible for the higher-order nonlinearity (particularly, third-order nonlinearity). In the case of non-resonant excitation (as in our current case), bound carriers at lower pump intensities play the dominant role, but at higher pump intensities, free carrier and two-photon absorption mechanisms are responsible for the nonlinearity. Searching for material with a strong third-order nonlinear response for non-resonant excitation is very important for applications in photonics. Hence, mixed perovskite films might be considered as a nonlinear material for non-resonant excitation conditions.

In this paper, we report on the generation of third harmonic emission and photoluminescence (PL) in perovskite nanocrystals (NCs) containing thin films excited by femtosecond laser pulses with a 1030 nm central wavelength at 50 kHz repetition rate. A blue shift of PL peaks at higher powers of the femtosecond laser pulses was analyzed. The morphology and composition of the thin films containing mixed perovskite were analyzed. We also studied the enhancement of the coherent third harmonic generation in 0.5 μm-thick perovskite film. The emission spectra of (FAPbI_3_)_1−x_(MAPbBr_3_) x, with a constant mole ratio x that equals 0.08 under a laser excitation power ranging from 0.2 W to 1.0 W, was investigated. The nonlinear refractive index and nonlinear absorption parameters of the sample were analyzed using Z-scan measurements.

## 2. Experimental Arrangements

We used broadband 38 fs laser pulses (AFSUFFL-300-2000-1030-300; Active Fiber Systems GmbH) at central wavelength of λ = 1030 nm for analyzing the generation of the 3rd harmonic (λ/3 = 343 nm) generation and incoherent PL signal in the thin film coated on the surface of quartz substrate by spin coating machine. The quartz substrate was chosen due to its transparency in the ultraviolet range of spectrum and the highest optical strength at the strong field of the laser pulses. The (FAPbI_3_)_0__.__92_(MAPbBr_3_)_0__.__08_ perovskite film was prepared in a nitrogen-filled glove box by a one-step method reported in [19]. PbI_2_ (anhydrous, 99.99%, Xi’an Polymer Light Technology Corp., Xi’an, China), PbBr_2_ (anhydrous, 99.99%, Xi’an Polymer Light Technology Corp.), methylammonium chloride (MACl) (anhydrous, 99.99%, Great Solar), methylammonium bromide (MABr) (anhydrous, 99.99%, Great Solar), and formamidinium iodide (FAI) (anhydrous, 99.99%, Great Solar) were used as precursors. The solvents used were DMF (anhydrous, 99.8%, Sigma-Aldrich) and DMSO (anhydrous, 99.9%, Sigma-Aldrich). Firstly, the precursor solution was prepared by dissolving lead iodide (1.5 M PbI_2_), lead (II) bromide (0.1 M PbBr_2_), methylammonium bromide (0.1 M MABr), methylammonium chloride (0.5 M MACl), and formamidinium iodide (1.2 M FAI) in anhydrous dimethyl sulfoxide-dimethylformamide (DMSO-DMF) = (1:4 *v*/*v*). After stirring for 4–5 h, 45 µL of the prepared solution was deposited on the substrate and spin-coated at 1000 rpm for 10 s, followed by 6000 rpm for 20 s. During the second step, 200 µL of anhydrous chlorobenzene was dynamically dispensed onto the center of the film 10 s before the end of the spin-coating. The substrate was eventually annealed at 150 °C for 20 min.

**Figure 1:**
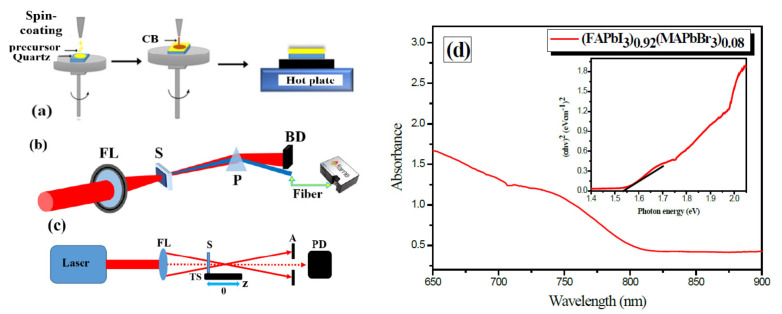
(**a**) Schematic diagram of the deposition steps for the perovskite NCs thin film on the surface of quartz substrate. (**b**) Experimental setup: focusing lens (FL/400 mm), sample (S), calcite prism for dispersion of the main harmonics and their different orders (in particular, the 3rd harmonics), beam dump for dumping main harmonics, silver-coated mirror to direct the 3rd harmonic to the fiber spectrometer. (**c**) Experimental scheme for Z-scan measurements. LASER, femtosecond fiber laser; FL, focusing lens; S, sample; TS, translating stage; A, aperture; PD, photodiode. (**d**) Absorption spectra of the thin film containing mixed perovskite NCs. Inset: the Tauc-plotted absorption spectra of the sample.

The femtosecond laser pulses were focused by 400 mm focusing lens on the surface of the sample (Figure 1b). The generation of 3rd harmonic in the perovskite sample was analyzed based on the third-order nonlinear response of the NLO materials [14]. During our studies, the sample was installed before the focal plane of the focusing lens of 400 mm focal length. This configuration allowed for the avoiding of the optical breakdown of the sample when high intensities of the 38 fs laser pulses are applied.

The beam spot size on the sample target was 200 μm and, by measuring the energy of the focused femtosecond laser pulses, the laser intensity was determined. The maximal intensity of the fundamental laser pulses (*λ* = 1030 nm) was equal to 3.0 × 10^12^ W cm^−2^ for 2 W average power of the laser pulses at a 50 kHz repetition rate. The laser intensity was controlled by changing the average power of the laser pulses using combination of a half-wave plate and a thin-film polarizer, which were installed before the focusing lens (FL). The dispersion of the fundamental *λ* = 1030 nm laser beam, which is 3rd harmonic (*λ* = 343 nm), was analyzed using a calcite prism. Fiber spectrometer (Flame, Ocean Optics, Orlando, FL, USA) was used for measuring the 3rd harmonic and PL signals, as presented in Figure 1b. In the case of the PL signal, the fiber of the spectrometer was installed close to the sample to allow for maximum detection of the PL signal generated from the sample.

The morphology and topography of the thin film were analyzed using a scanning electron microscope (SEM, VEGA3, TESCAN, Brno, Czech Republic) and atomic force microscope (AFM, Nanomagnetics, UK). The absorption spectrum of the sample was taken with a fiber spectrometer (Flame, Ocean Optics) (Figure 1d) [20]. The optical bandgap of the perovskite thin film was estimated with Tauc plot (inset on Figure 1d). The bandgap energy of the mixed perovskite NCs thin film was defined to be 1.50 eV, which corresponds to the value of the perovskite of this composition [5,6]. The thickness of the sample was also studied using 3D Profilometer (Profilm3D, Filmetrics, Unterhaching, Germany), and it was found to be 0.5 μm. This thickness of the sample can be considered as an effective thickness of the thin perovskite film.

The standard Z-scan technique was employed for determining the third-order nonlinear optical parameters of the thin film containing mixed perovskites [21]. In this technique, two schemes are commonly applied. The closed aperture (CA) scheme allows for determination of the sign and magnitude of nonlinear refractive index (γ); and the open aperture scheme (OA) is used to measure the sign and magnitude of nonlinear absorption coefficient (β) (Figure 1c). In the closed aperture (CA) scheme, we used a 400 mm focal length lens, translating stage to move sample with respect to the focal plane of the focusing lens, aperture (A), and photodiode (PD). In the case of open aperture (OA) Z-scans, the aperture was removed to collect all propagated radiation by a calibrated PD. Z-scan curves of a normalized transmittance were recorded during the scanning of the sample along the *z*-axis of the focused laser beam.

The fundamental harmonic (λ = 1030 nm) of the femtosecond laser (pulse duration 38fs, pulse repetition rate 50 kHz) was focused using a 400 mm focal length spherical lens. The beam waist diameter was 60 μm. Correspondingly, the maximal intensity of the laser beam was estimated to be 4.2 × 10^11^ W cm^−2^. The Z-scan setup was calibrated using a 1 mm-thick fused silica plate.

## 3. **Results and Discussion**

**Figure 2:**
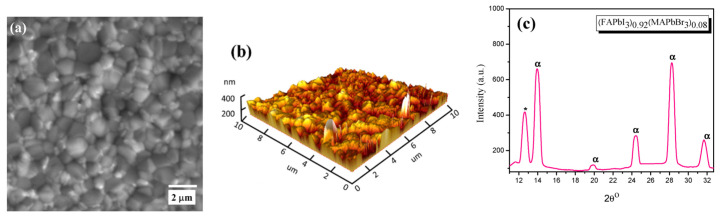
(**a**) SEM and (**b**) AFM microphotography and (**c**) XRD of the perovskite thin film. The homogenous distribution of the nanocrystals on the surface of the substrate is presented by AFM 3D image. XRD spectra peaks for (FAPbI3)0.92(MAPbBr3)0.08 deposited on the quartz substrate. The crystallinity structures were supported by XRD measurement of the thin films.

Figure 2 shows the morphology of the samples characterized using the electron and atomic force microscopes. It can be seen from Figure 2a that the surface of (FAPbI_3_)_0.92_(MAPbBr_3_)_0.08_ deposited on quartz and annealed at 150 °C for 20 min shows a uniform morphology and well-grown crystallites. The AFM characterization results are presented in Figure 2b, where the surface 3D morphology is quite homogenous for such optical studies of the sample. From the SEM and AFM images, the grain size and roughness of the surface can be inferred, and an estimation of the average sizes of grain was 400 nm, with a 200 nm roughness on the surface of the quartz substrate.

Figure 2c shows the X-ray diffraction (XRD) patterns for the perovskite layer on a quartz substrate, which showed the crystalline structure of these species. As expected, for the perovskite structure, the characteristic peak (α) is indexed at $2\theta \approx 14.1^{{}^{\circ}}$. An unreacted PbI_2_ in the precursor is indicated by the peak (δ) at $2\theta \approx 12.8^{{}^{\circ}}$. PbI_2_ could act as a defect that traps charge carriers and then directly yields a reduction in the purity of the perovskite as a device by blocking the passivation at the interfaces between perovskite and the transporting layers [22]. A tetragonal structure was reported for (FAPbI_3_)_0.92_(MAPbBr_3_)_0.08_ with a space group of 14 cm, with the following lattice parameters: a (Å): 8.8550, b (Å): 8.8550, c (Å): 12.5350), which will prohibit the generation of a second harmonic from the probing laser beam, rendering it as a noncentrosymmetrical structure with second-order susceptibility [23]. As shown in Figure 2c, the characteristic peaks could be identified easily, and the structure is dominated by FAPbI_3_. The signs of δ and α correspond to PbI_2_ and (FAPbI_3_)_0.92_(MAPbBr_3_)_0.08_, respectively.

**Figure 3:**
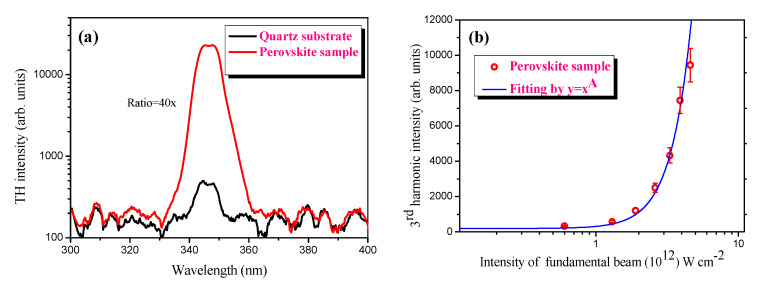
(**a**) Comparative log−scale spectra of third harmonic signal generated from pure quartz (black solid curve), and mixed perovskite (red-solid curve) using 1030 nm 38 fs laser pulses. (**b**) Log−scale dependence of third harmonic signal generated in the mixed perovskite on the intensity of the fundamental laser beam. The cubic (A = 3) dependence of the third harmonic intensity on the intensity of the fundamental laser pulses was obtained.

For the optical studies, the homogeneity of the deposited thin film on the surface of the substrate allowed for the generation of homogeneously distributed third harmonic and PL signals. We analyzed the third harmonic generation of femtosecond laser pulses in the thin films containing perovskite. The frequency conversion of the broadband femtosecond laser pulses was recorded in the ultraviolet range of the spectrum at a wavelength of 345 nm. The comparative spectrum of the third harmonic signal between the perovskite NCs film and the quartz substrate is presented in Figure 3a. The 40× enhanced coherent third harmonic signal was detected in the thin film containing mixed perovskite compared to quartz. We have maintained a moderate intensity (2.5 × 10^12^ W·cm^−2^) for the driving femtosecond laser pulses in order to avoid the breakdown of the sample deposited on the surface of the quartz substrate. In our study, the maximal fluence of the driving laser pulses had a value of around 0.1 J·cm^2^ for the generation of TH in the thin film containing perovskite NCs, which is significantly less than the ablation threshold of the pure quartz substrate. Correspondingly, we did not observe craters on the surface after irradiation by driving laser pulses at the same fluence. Moreover, the range of intensities of the driving laser pulses was even less than the ablation threshold of the perovskite thin films. Therefore, no damage in the sample or surface of the quartz substrate was observed after irradiation with femtosecond laser pulses. The ablation threshold of the perovskite NCs containing the thin film was estimated to be 0.23 J·cm^−2^.

The nano-sized structure of the mixed perovskite material played an important role in the generation of the third harmonic of 1030 nm femtosecond laser pulses. The evidence of these nanostructured species was demonstrated by SEM and AFM images of the thin film. The third harmonic signal intensity and its dependence on the intensity of driving pulses in the thin film of perovskite are shown in Figure 3. As expected, a cubic dependence (Figure 3b) of the third harmonic signal on the power of the femtosecond laser pulses is obtained. It is worth mentioning that the bandwidth of the signal of third harmonics presented in Figure 3a is defined by the bandwidth of the driving pulses. In our case, the driving 38 fs laser has a wide range spectrum from 900–1050 nm.

The intensity of a generated third harmonic in the case of an isotropic nonlinear medium can be estimated according to [23]:

(1) $$I_{3\omega }=\gamma^{2}l^{2}I_{10}^{3}\exp(-6k_{1}r^{2}/b)\frac{\sin^{2}\Delta (l,r)}{\Delta^{2}(l,r)},$$

where *γ* = 24π^3^χ^(3)^(−3ω; ω, ω, ω)/(*n*_1_^3/2^*n*_3_^1/2^*c*λ_1_), Δ(*l, r*) = 2*b*/*l* − *α* − *β*, *α* = 2*l*Δ*k* is the normalized phase-mismatching, *β* = 72π^3^*l*Δχ_k_*I*_10_*exp*(−2*k*_1_*r*^2^/*b*)/(*n*_1_^2^*c*λ_1_); Δ*χ*_k_ = *χ*^(3)^(−ω; ω, ω, −ω)/2 − *n*_1_*χ*^(3)^(−3ω; 3ω, ω, −ω)/*n*_3_ is the difference in Kerr-induced nonlinearities, responsible for refraction indices changes at the wavelengths of fundamental radiation and harmonic; λ*_i_*, *k_i_* and *n_i_* are the wavelengths, wave numbers and refraction indices on the frequency of *i*-radiation; *I*_10_ is the maximal intensity at the plane of the beam waist; *b* is the confocal parameter of the focused fundamental radiation; and *l* is the thickness of the nonlinear medium. Our studies of thin films showed qualitative similarity between the anticipated theoretical dependence and experimental data at larger energies of laser pulses (*I*_3ω_∞*I*_1ω_^3^). The theoretical calculations [24] have shown that, with a further increase in laser intensity, the power dependence of the third harmonic did not reach the saturation of the signal, and there was a notable decrease in the slope value. The positive sign of the nonlinear refractive index that we analyzed by Z-scan measurements also played an important role in improving the phase matching conditions at the wavelength of fundamental radiation and its third harmonic.

**Figure 4:**
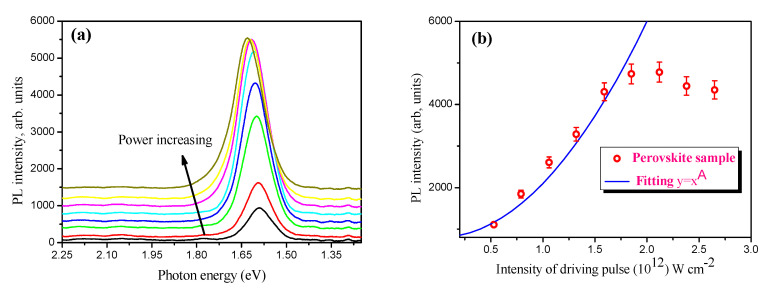
Power−dependent PL signal excited in the thin film mixed perovskite. (**a**) Power-dependent blue−shifted PL signal at the wavelength of 779.8 nm to 758.1 nm. The black arrow shows blue-shift of the PL peak. (**b**) Quadratic dependence (A = 2) of the PL emission integrated intensity on the intensity of the driving laser pulses at low excitation power is observed at 50 kHz repetition rate. The experimental data are presented by the red open circles and theoretical fitting is presented by the blue solid line.

Figure 4 presents the PL spectrum and the dependence of the PL intensity on the laser power (Figure 4a) and the intensity (Figure 4b) of the broadband femtosecond laser pulses in the perovskite NCs thin film. Continuously tunable optical band gap energy from 1.59 eV to 1.63 eV (758.1 nm < λ_PL_ < 779.8 nm) with different laser powers was observed (Figure 4a). Interestingly, a blue shift of the PL peak was observed by varying the average power of the fs-laser pulses (1030 nm) from 0.2 W to 1.0 W. As a result, the bandgap has broadened.

On the other hand, a third harmonic peak was detected at 350 nm (E_g_ = 3.54 eV). The nonlinear behavior of the PL intensity can be explained by the two-photon excitation of the free carriers of perovskite NCs. Meanwhile, the blue-shift of the maximum PL signal was observed at the high power of the femtosecond laser pulses. Figure 4a presents the variation of the blue-shift on the power of the femtosecond laser pulses.

The black arrow shows the blue-shift of the PL peaks in the power-dependent PL of the sample. The nonlinear behavior in power-dependent PL started at an intensity of 3.0 × 10^11^ W·cm^−2^ and was observed at an intensity of up to 1.75 × 10^12^ W·cm^−2^ of the probing laser pulses. A further increase in the intensity of the fundamental laser pulses led to the saturation of the intensity of PL generated in the thin film containing perovskite NCs. The saturation and blue-shift of the PL signals can be explained by the variation of the carrier’s density of perovskite at a high power of the fundamental laser pulses. These phenomena that are associated with slow hot carrier relaxation and the state-filling of band-edge states were analyzed by Fang et al. [25]. Additionally, PL saturation at higher pumping intensities could be due to exciton–exciton annihilation if multiple excitons are generated in the individual NCs, and such a fast recombination could lead to the rapid heating of the sample. However, such rapid heating might be possible under a continuous illumination of the sample with solar light, a CW laser, or laser pulses with much longer pulse durations [26,27]. In our work, we illuminated the sample with laser pulses that have a very short (~38 femtoseconds) duration, which is much shorter than the relaxation time of the exciton–exciton interactions. Another possible explanation for the blue-shift could be attributed to reabsorption on the blue side of the emission by the excitonic absorption peak (the reabsorption is caused by increasing the carrier density in the excited state in the perovskite nanocrystal). In our case, the measurement of the PL signal was performed at the backside of the sample, where the reabsorption of the blue side (shorter wavelengths) of the PL is possible. As the excitons diffuse across the sample, less light is reabsorbed and a blue-shift is observed [25,28,29].

Considering the fact that we are using ultrashort driving pulses (~38 fs duration) the possibility of photodegradation is unlikely. In the power-dependent PL spectra, we added a background signal to each PL curve to clearly demonstrate the blue-shift of PL spectra. This is because, at the maximal power of the driving fs pulses, we observed a small decrease in the intensity of PL, as shown in Figure 4b. Previous studies have shown that the photodegradation of perovskite thin films is possible when irradiating with continuous waves for a long time, or irradiating with visible fs laser pulses [30]. The photochemical degradation of the perovskite films was observed upon irradiation with femtosecond pulses at 532 nm, and the depth of photodegradation decreased in the perovskite films protected with a PMMA polymer layer. In our case, we used femtosecond infrared driving pulses, where the absorbance of the sample is negligible and the photodegradation of the perovskite thin film can be ignored.

**Figure 5:**
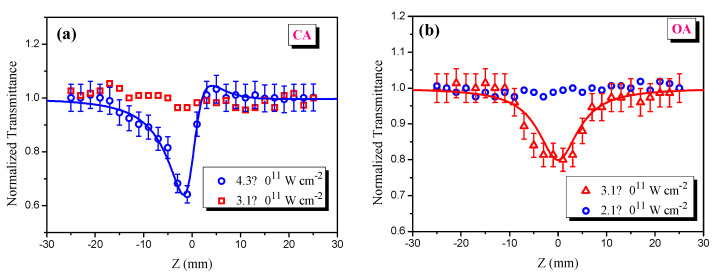
(**a**) CA and (**b**) OA Z−scan curves of the thin film mixed perovskite at 1030 nm wavelength laser pulses. In the case of CA, the intensity of the probing laser beam was ~4.3 × 10^11^ W·cm^−2^, where self-focusing effect (blue open circles) was observed. For probing beam with intensity ~3.1 × 10^11^ W·cm^−2^, the normalized transmittance did not change (red open squares). In the case of OA, the intensity of the probing laser beam was 3.1 × 10^11^ W·cm^−2^, where TPA effect (red open triangles) was obtained. The normalized transmittance (blue open circles) did not change at the intensity of 1.2 × 10^11^ W·cm^−2^ of probing laser beam.

The nonlinear behavior of the PL in the perovskite at the same intensity is consistent with our results obtained by Z-scan measurements (Figure 5b). Figure 5 shows the normalized transmittance of Z-scan curves. In both CA (closed aperture) and OA (open aperture) schemes of the Z-scan at relatively low intensities of the laser pulses, the normalized transmittance of the sample did not demonstrate any change. By increasing the intensity of the laser pulses, positive nonlinear refraction and TPA processes were observed in the mixed perovskite thin films. Our results on the PL based on the TPA can also be supported by analyzing the TPA process and estimation of the TPA coefficient of the thin perovskite NCs film by using an OA scheme of Z-scan measurements.

Using the Z-scan technique [31], the nonlinear optical characteristics of the thin perovskite film deposited on the substrate of quartz plate can be investigated. By defining the relative coordinate *x = z/z*_0_, *z*_0_ being the Rayleigh length, the dependence of the normalized transmittance *T*(*x*) in the case of the closed aperture (CA) Z-scan can be written as [32]:

(2) $$T\left(x\right)=1-\frac{4x}{\left(x^{2}+9\right)\left(x^{2}+1\right)}∆\Phi_{0}+\frac{2(x^{2}+3)}{\left(x^{2}+9\right)\left(x^{2}+1\right)}∆\Psi_{0}$$

where *z*_0_ *=* 0.5 *kw_o_^2^,* Δ*Φ*_0_ *= kγL_eff_I*_0_, Δ*Ψ*_0_ = *βI*_0_*L_eff_*/2, *k =* 2*π/λ* is the wave number, *w_o_* is the beam waist radius of the focused radiation, *I*_0_ is the intensity of the probe beam at the focal plane of the focusing lens, *γ* is the nonlinear refractive index, *β* is the nonlinear absorption coefficient, *L_eff_ =* [1 *− exp (−α*_0_*L*)]/*α*_0_ is the effective length of the nonlinear medium, *L* is the sample thickness, and α is the linear absorption coefficient of suspension. The nonlinear refraction index and nonlinear absorption coefficient were determined by the theoretical fitting of experimental data using Equation (2). The error bars for the determination of the absolute values of nonlinear absorption and refraction coefficients were estimated to be ±20% due to uncertainty in the measurements of the beam waist of the focused probe beam. The value of the measured nonlinear refractive index was equal to *γ* = +1.4 × 10^−12^ cm^2^·W^−1^, and the value of the TPA was equal to *β* = +7.0 × 10^−6^ cm·W^−1^. Due to the contribution of the strong TPA coefficient, the quadratic dependence of the emission intensity on the intensity of the 1030 nm probe pulse was observed (see Figure 4b). The results of the nonlinearity of the mixed perovskite sample are revealed to be comparably large, with nonlinear absorption in organic–inorganic perovskite thin films, where the values are in the upper range of the reported data for TPA absorption coefficients (10^−6^ to 10^−8^ cm·W^−1^ [33,34,35]). The value of the nonlinear refractive index can reach up to 10^−9^ to 10^−11^ cm^2^·W^−1^ in the perovskite films at the resonant excitation by femtosecond laser pulses [18]. In our case, the relatively high nonlinear refractive index played an important role in the generation of strong third harmonics of 1030 nm laser pulses. The self-focusing based on the positive nonlinear refractive effect of laser pulses in the sample can increase the refractive index, which may improve the phase-matching conditions slightly for both the driving pulse (1030 nm) and its third harmonic (343 nm). In particular, mixed (FAPbI_3_)_0_._92_(MAPbBr_3_)_0_._08_ with a bandgap energy of 1.50 eV might demonstrate strong TPA compared to single-cation perovskites MAPbBr_3_ (1.54 eV) due to the smaller bandgap. This can be analogous to the *β~*(*E_g_*)*^−3^* dependence of the TPA coefficient on the bandgap, as found with the Cu and CuO nanoparticles of variable band gaps [36,37]. Moreover, the nanostructured morphology of the mixed perovskite NCs can also play an important role in the nonlinear response of the medium containing NCs [38], nanowires [39], and quantum dots of perovskites [40]. These forms of thin films containing perovskites allow for the tunable control of their nonlinear optical responses for a wide range of the spectrum, which enhances their applicability in photonics as passive modulators with saturable absorptions.

Temperature-dependent red-shifted PL for two different perovskite NCs was discussed in Ref. [41]. The CsPbC_l1.5_Br_1.5_ NCs, deposited on heat-treated glass at 530 °C, exhibited strong TPA and three-photon absorption coefficients compared to the perovskite NCs at higher temperatures. Such a shift was attributed mainly due to the better crystallization properties and the increase in the crystal grain size. TPA-based PL in the FAPbBr_3_ perovskite NCs was supported by z-scan measurements using 800 nm fundamental laser pulses [42], where the central wavelength of the PL was observed at a 536 nm wavelength. In our case, we note that, in the intensity-dependent PL for the mixed (FAPbI_3_)_0.92_(MAPbBr_3_)_0.08_ perovskite NCs, the slope of the curve gives a value of 2 (Figure 4b), which implies that the emission intensity has a quadratic dependence on the intensity of the driving laser pulses. This validates the TPA measurements from our Z-scan studies that are conducted with the 50 kHz laser. It can then be possible that the saturation of the PL signal occurs due to the heating of the sample when higher powers of the driving 50 kHz laser pulses are applied, where the temperature-dependent carrier’s density variation can be triggered.

Despite the extensive research on the third-order nonlinear response of thin films containing nanostructures, the conversion efficiency of the third harmonic remains at a low level (10^–5^–10^–6^) [43,44]. For example, the THG conversion efficiency has reached values of up to 1.2 × 10^–6^ by the enhanced nonlinearity in the silicon metasurface due to a high Q-factor Fano resonance. Our current study focuses on the nonlinear optical properties of the thin film containing perovskite nanocrystals when irradiated by 38 fs laser pulses with a 1030 nm central wavelength. Compared to the quartz substrate, the thin film containing perovskite nanocrystals exhibited a 40× enhancement in the TH signal, which is attributed to the giant 2PA and moderate nonlinear refraction index in the thin film containing perovskite NCs.

Our findings show that the strong third-order nonlinear response of the mixed perovskite NCs thin films makes them useful as a nonlinear medium for the generation of the high-order harmonics [45]. Moreover, this response of the thin films containing perovskite nanocrystals is similar to the one observed with metal oxides, which also show high-order nonlinear responses via the generation of the effective high harmonic generation of ultrashort laser pulses. In particular, Gholam-Mirzaei et al. have reported a direct application of thin films containing nanostructured ZnO materials that have demonstrated the generation of high-order harmonics of mid-IR laser pulses [46]. A boost in the harmonic yield by a factor of two and the spectral broadening of above-gap harmonics, compared to longer driving pulses, were generated from ZnO NCs. Furthermore, the application of the laser-induced plasmas (LIPs) containing perovskite nanocrystals was considered as a nonlinear medium for the generation of high-order harmonics of 800 nm, 36 fs laser pulses [47]. It was shown that the enhanced emission of harmonics from the LIPs of these pristine, Ni-doped CsPbBr_3_ colloidal 2D NCs might be useful in attosecond spectroscopic studies via the generation of high-order harmonics. Moreover, the laser- induced plasmas on the surface of thin films containing gold nanoparticles [48] and complex copper oxide nanoellipsoids [37] have generated strong high-order harmonics of 800 nm femtosecond laser pulses. Hence, thin films containing nanostructured material of perovskite, metal nanoparticles, etc., can be an efficient nonlinear medium that could have many applications within strong-field nonlinear optics.

## 4. Conclusions

In summary, we reported the generation of coherent third harmonics of broadband femtosecond laser pulses in thin films containing perovskite deposited on the surface of a quartz substrate. A comparative analysis of the coherent third harmonic generation in the thin film containing perovskite NCs has demonstrated a 40× enhancement of the third harmonic signal compared to the signal generated in the pure quartz substrate. The two-photon absorption-based incoherent PL signal was also observed for a range of powers of the driving laser. Due to the variation in the density of the carriers, blue-shift and saturation of the PL signals were recorded. The third-order nonlinear optical response of (FAPbI_3_)_0.92_(MAPbBr_3_)_0.08_ perovskites showed a cubic dependence on the intensity of the fundamental laser radiation. The positive nonlinear refractive index (γ = +1.4 × 10^−12^ cm^2^·W^−1^) has shown a significant role in improving the phase-matching condition of interacting pulses by generating a strong third harmonic. The giant TPA-assisted PL peak was monitored and a blue shift of the PL was obtained when high intensity laser pulses are used. The giant TPA absorption coefficient was estimated to be β = +7.0 cm·MW^−1^ at a 1030 nm wavelength of laser pulses. The presented findings on the non-linear process of the mixed perovskite make them a strong candidate for many promising applications and opens the door for new applications, such as the development of efficient frequency conversion systems, optical switching, and the modulation of light to control the THG efficiency [49]. Moreover, the extracted optical properties can provide insights on the crystallinity properties of the perovskite crystals, using, for example, time-resolved optical traces and generating a cluster analysis, which can be used to develop novel optoelectronic devices [50].

## Data Availability

Data underlying the results presented in this paper are not publicly available at this time but may be obtained from the authors upon reasonable request.
